# Comparison of Aerosol Stability of Different Variants of Ebola Virus and Marburg Virus and Virulence of Aerosolised Ebola Virus in an Immune-Deficient Mouse

**DOI:** 10.3390/v14040780

**Published:** 2022-04-09

**Authors:** Sophie J. Smither, Lin S. Eastaugh, Mark S. Lever

**Affiliations:** Microbiology and Aerosol Sciences Group, CBR Division, Defence Science and Technology Laboratory (Dstl), Porton Down, Wiltshire, Salisbury SP4 0JQ, UK; lseastaugh@dstl.gov.uk (L.S.E.); mslever@dstl.gov.uk (M.S.L.)

**Keywords:** Ebola virus, Marburg virus, aerosol, stability, mouse model, virulence, variants, isolates

## Abstract

During outbreaks of virus diseases, many variants may appear, some of which may be of concern. Stability in an aerosol of several Ebola virus and Marburg virus variants was investigated. Studies were performed measuring aerosol survival using the Goldberg drum but no significant difference in biological decay rates between variants was observed. In addition, historic data on virulence in a murine model of different Ebola virus variants were compared to newly presented data for Ebola virus Kikwit in the A129 Interferon alpha/beta receptor-deficient mouse model. Ebola virus Kikwit was less virulent than Ebola virus Ecran in our mouse model. The mouse model may be a useful tool for studying differences in virulence associated with different variants whereas aerosol stability studies may not need to be conducted beyond the species level.

## 1. Introduction

The COVID-19 pandemic has shown how new variants of a virus may be a cause for concern and may impact infection and pathogenesis or lead to immune escape. New variants may affect the spread of the virus and the efficacy of vaccines as well as hospital admissions and severity of disease [1,2]. Changes in transmission rates can influence control measures such as the wearing of masks and maintaining safe distances and also can play a key role in determining levels of restrictions and the time to ‘return to normal’.

Filoviruses cause severe disease and remain a problem with multiple outbreaks having occurred in Africa since the viruses were first identified [3,4,5]. The viruses responsible for most outbreaks and highest case fatality rates are Ebola virus (EBOV) and Marburg virus (MARV). In 2021, there were two outbreaks of Ebola virus disease, in the Democratic Republic of the Congo (DRC) and in Guinea, [3,6], with the DRC being Ebola-free for only a few months [3]. 2021 also saw a case of MARV infection in Guinea which is the first time MARV has been identified in West Africa [7]. Although there has not been a large MARV outbreak for over a decade, there have been small numbers of cases every few years, the last in 2017, and the largest two outbreaks to date, in DRC (1998–2000) and Angola (2004–2005) have resulted in extremely high case fatality rates of 83% and 90% respectively [5]. EBOV and MARV are both on the WHO high priority pathogens list [8] which means both are among those “diseases that pose the greatest public health risk due to their epidemic potential and/or whether there are no or insufficient countermeasures” and are therefore in need of urgent Research and Development action.

Each outbreak of EBOV or MARV is typically associated with the emergence of a different variant and multiple isolates with the result that many variants and isolates exist.

Different institutes may have different viruses available for research and different criteria for which virus should be used for the standardization of protocols and procedures [9]. Our definition of isolate and variant and the nomenclature used for filoviruses are based on that described by Kuhn et al in an effort to achieve standardization [10]. A variant ‘differs in its genomic consensus sequence from that of a reference filovirus’ whilst an isolate is ‘an instance of a particular virus’ [10].

Although genome sequencing has rapidly improved over the years, even once the causative agent is known it can take time to get a full sequence and understand genetic relatedness. It may not always be possible to acquire and import an isolate from the latest variant available, for conducting studies, and doing so might take some time, particularly if adaptation is required, for example in the development of animal models. When developing and designing medical countermeasures or putting in control measures for a new outbreak, researchers may choose to work with a recent isolate from that particular outbreak, use pre-existing isolates from earlier outbreaks that are readily available and may be more fully characterized, or, researchers may decide to work with a prototype or type strain as a representative virus for that species.

This paper describes the results of investigations comparing the aerosol stability of two different variants of EBOV, the Kikwit variant from 1995 recommended by the Filovirus Animal Nonclinical Group (FANG) for standardizing studies [11] and the Makona variant (C07 isolate) from the largest EBOV disease outbreak to date which started in West Africa in 2013. In addition to aerosol stability, to understand transmissibility and environmental durability, both these variants, alongside an EBOV from the original outbreak in 1976 (the Ecran isolate of the Yambuku variant), have been aerosolized for exposure to mice to look at virulence. We present previously unpublished data on EBOV-Kikwit in mice and compare it to our previously published observations on EBOV-Ecran [12] and EBOV-Makona [13].

In general, MARV is less studied than EBOV. We present aerosol survival comparison data between an original outbreak isolate from 1967 (Popp), and the well-characterized and FANG recommended variant (Angola), from the largest outbreak to date of MARV which occurred in 2005. These MARV isolates have also been tested in mice for virulence, but only MARV-Popp was tested by the aerosol route so no comparison data is shown here.

This work will help inform on the relative aero stability and virulence of different variants and isolates. Studying aerosols is important due to the possibility of deliberate or accidental release or generation of aerosolized virus. Survival and stability studies may help in predicting how a future isolate or variant might perform, based on phylogenetic similarities or differences and thus this data might inform risk assessments and control measures. Understanding the virulence of different variants and isolates in a small animal model may help inform which isolates could be used for vaccine or therapy screening and may help in predicting the severity of new variants. Differences in virulence may also point to targets for intervention.

## 2. Materials and Methods

### 2.1. Viruses

Ebola virus/H.sapiens-tc/COD/1976/Yambuku-Ecran, referred to as Ebola virus E718 in some earlier work [14] and hereafter referred to as EBOV-Ecran and Ebola virus/H.sapiens-wt/GIN/2014/Makona-C07, hereafter referred to as EBOV-Makona and Marburg virus/H.sapiens-tc/GER/1967/Hesse-Poppinga, hereafter referred to as MARV-Popp were provided by Public Health England, UK. Ebola virus/H.sapiens-tc/COD/1995/13625-Kikwit, hereafter referred to as EBOV-Kikwit and Marburg virus/H.sapiens-tc/AGO/2005/Angola-200501379, hereafter referred to as MARV-Angola were provided by the Public Health Agency of Canada. For a summary of viruses described in this paper, see Table 1. All three EBOV isolates used in the work described herein and MARV-Angola have been sequenced and identity confirmed (Table 1). MARV-Popp has been partially sequenced and confirmed as the Marburg virus (personal observation).

### 2.2. Growth and Enumeration

All virus growth and enumeration assays were performed in Vero C1008 cells (ECCAC Cat. No. 85020206) maintained in Dulbecco’s minimum essential tissue culture medium (TCM) (Sigma) supplemented with 2% Fetal Calf Serum, 1% L-glutamine, and 1% Penicillin/streptomycin (all Sigma). All growth and incubation were at 37 °C and 5% CO_2_. Enumeration was via the 50% tissue culture infectious dose (TCID_50_) assay. Historically, the TCID_50_ assay was performed as described previously [15] with cells being stained and fixed prior to scoring for cytopathic effects by eye. More recently (2015 onwards so for all aerosol survival data presented here), the assay has been simplified with no staining or fixing, and plates being scored by observation of cytopathic effects under the microscope. All calculations of the endpoint are via the method of Reed and Muench [16].

### 2.3. Aerosol Decay Studies

Studies to determine aerosol stability were performed in the rotating 40 L Goldberg drum as described recently [17,18] within Advisory Committee on Dangerous Pathogens (ACDP) Containment Level 4 (BSL4 containment). Briefly, the AeroMP (BiAera Technologies, LLC) and a 3 jet Collison nebulizer (known to produce particles in the range of 1–5 µm) [19] were used to generate and condition an aerosol at 30 L/min and 50% relative humidity. Impinger sampling was at 4 L/min for 1 min using midget impingers and these were collected into 3 mL TCM. For all viruses, a volume of 8 mL was sprayed for 5 min to fill the drum, and the contents were then mixed for 2 min prior to the first (“T0”) sample being taken. Impingers were collected at various points over 90 min. Sprays were performed on three separate occasions, and, at each time-point, impingers were assayed in triplicate making a total of 9 titers for each virus per time-point. All drum experiments were performed in the dark, at ambient temperature (19–21 °C) and relative humidity (42–57%).

### 2.4. Animal Studies

Animal studies were performed in accordance with the UK Scientific Procedures Act (Animals) 1986 and UK Codes of Practice for the Housing and Care of Animals Used in Scientific Procedures, 1989. Six-to-nine-week-old A129 Interferon alpha/beta receptor-deficient mice (IFN-α/β R^−/−^) (B & K Universal, UK) were housed in cages of 2–6, depending on the study and sex mix available. Mouse weight at the time of challenge was 17–23 g for females and 22–28 g for males. Group totals were 5, 6, or 10, split equally male/female; or 2 + 3 of either sex in the case of *n* = 5. All-female groups were used when available. Mice were housed within ventilated cages in a half-suited isolator under BSL4 containment and had *ad libitum* access to food and water as previously described [12,13].

Aerosol challenges were as described previously [12]. Briefly, mice were exposed to small particle aerosols of virus for 10 min and impinger samples, collected at 12 L/min into All Glass Impingers, were taken for 1 or 2 min during the middle of the aerosol challenge. Calculations of retained dose based on impinger samples enumerated by TCID_50_, were as previously described [12,13]. An average mouse breathing rate of 0.02 L/min determined by Guyton and colleagues [20]) was assumed and it was also assumed that 40% of the inhaled dose was retained in the body [21].

For intraperitoneal (IP) challenge, mice were challenged with 100 µL via the IP route. All dilutions for challenges were made in TCM. After challenge, mice were weighed daily and observed at least twice daily for clinical signs or mortality. Mice were culled upon reaching the humane end-point (typically >20% weight loss or immobility).

Murine studies with EBOV-Ecran, EBOV-Makona, and MARV-Popp have been previously described [12,13].

The mouse studies not previously described were two studies with EBOV-Kikwit. For the first EBOV-Kikwit study, groups of 5 mice (mixed-sex) were challenged with a range of doses of EBOV-Kikwit by the aerosol or IP route. In the second EBOV-Kikwit study, groups of 10 mice (mixed sex) were challenged with a range of doses by the IP route only.

### 2.5. Statistical Analysis

For analysis of aerosol data, impinger counts of the virus at each time-point were adjusted to account for the dilution effect of removing 4 L per sampling time-point, and the mean count per time-point for each of the three runs was determined. Linear regression was performed on the natural log (ln)-transformed data. Analysis was performed in GraphPad Prism 8.0.1. Log-rank analysis was performed on mouse survival data.

## 3. Results

### 3.1. Different EBOV and MARV Variants Show Similar Biological Decay Rates in Aerosols

**Figure 1:**
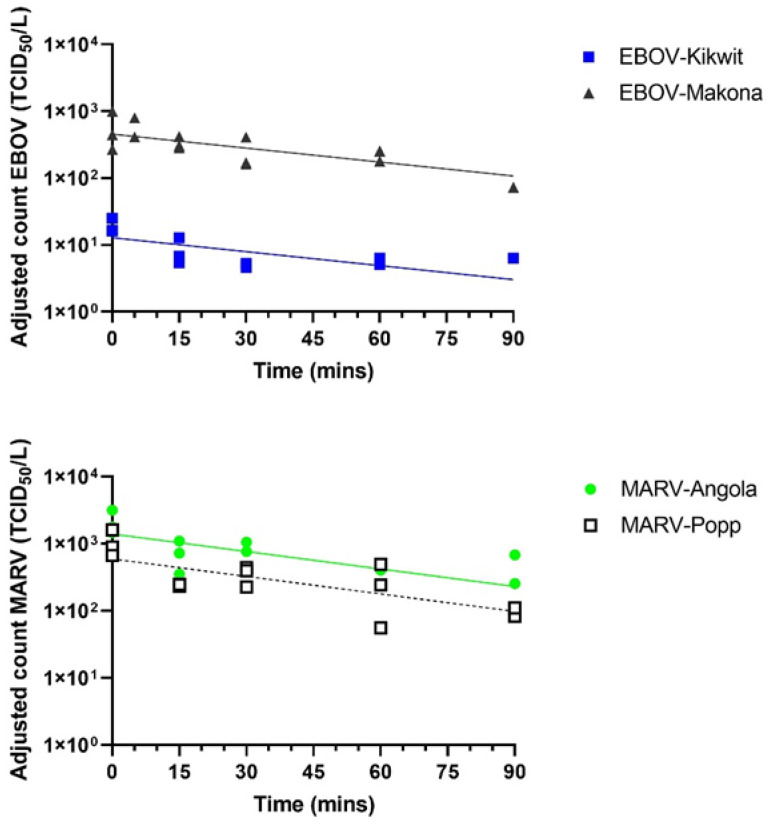
Aerosol survival of different variants of EBOV and MARV. Filoviruses were aerosolized and held as a dynamic aerosol in the Goldberg drum. Impinger samples were taken at various time-points and enumerated for viable viruses by TCID_50_ assay in triplicate. Each virus was assessed on three separate occasions. Graphs show the adjusted mean count for each run (as individual data points) for two EBOV variants (**top**) and for two MARV variants (**bottom**). Best fit lines were fitted to the data using linear modeling.

MARV-Angola and MARV-Popp and EBOV-Kikwit and EBOV-Makona were all aerosolized and the biological decay rate over time was measured using the Goldberg drum (Figure 1). All four viruses were aerosolized on three separate occasions and had similar starting titers (Table 1), however, there was a consistently low recovery of EBOV-Kikwit. Decay was observed for all viruses, with viable virus still detected after 90 min for both MARV-Angola and MARV-Popp and for EBOV-Makona. For EBOV-Kikwit, reduced stability to the aerosolization process meant the amount of virus recovered was very close to the limit of detection. Viable EBOV-Kikwit could still be detected in all three runs at 30 min, in two of the three runs at 60 min but only in one of the three runs at 90 min.

Linear model analysis was performed on the natural log-transformed mean titer of each run. The slope of the curve was determined and compared. For MARV-Angola the slope was −0.019 and for MARV-Popp the slope was −0.021. There was no significant difference between the biological decay curves for the two Marburg viruses. For EBOV-Makona the slope of the curve was −0.019 and for EBOV-Kikwit it was estimated at −0.013 and again there was no significant difference between the decay curves for the two EBOV variants. A mean decay rate of 1.6% min^−1^ for EBOV translates to a half-life of approximately 43 min whilst a mean decay rate of 2.0% min^−1^ for MARV equals a half-life of approximately 35 min.

### 3.2. EBOV-Kikwit Shows Lethality in the Mouse Model at High Doses Only

**Figure 2:**
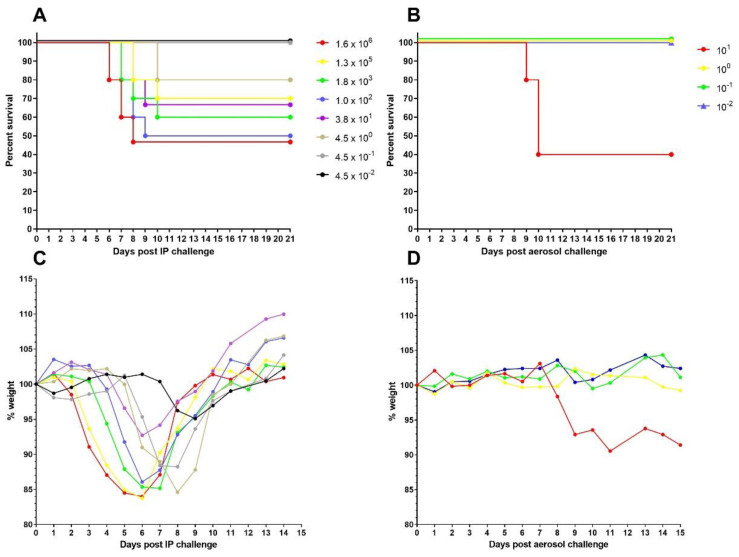
Survival and weight loss in A129 Interferon alpha/beta receptor deficient mice challenged with EBOV-Kikwit. Groups of mice were challenged with a range of doses of EBOV-Kikwit (shown by different colors) by the IP (**A**,**C**) or aerosol (**B**,**D**) route. Survival (**A**,**B**) and mean group weight (**C**,**D**) are shown. For weight loss, the mean group weight on the day of challenge was taken as 100%. For aerosol challenge, the results are from a single experiment with groups of 5 mice per dose. For IP challenge, the results are combined from two studies using the same stock of EBOV-Kikwit with groups of 5 in the first study and 10 in the second study resulting in an *n* = 15 for 10^6^ (red) and 10^2^ (purple) used in both experiments.

Mice were challenged with EBOV-Kikwit by the IP route on two separate occasions (data combined) or by the aerosol route once and monitored for 21 days (Figure 2). Clinical signs observed (as previously reported) were ruffled coat, hunched posture, reduced mobility, and closed eyes. Mice showed a dose-response to IP challenge with EBOV-Kikwit but even the highest dose administered, which was tested twice (mean dose = 1.6 × 10^6^ TCID_50_) only caused around 50% lethality (8 of 15 mice total) with animals succumbing from day 5 onwards (Figure 2A). Doses between 10^5^ and 10^2^ were tested once (*n* = 10 per group) and resulted in 3–5 mice per dose succumbing (Figure 2A). The -4 dilution was tested twice (mean dose = 38 TCID_50_) and resulted in a combined total of 5 of 15 mice succumbing. Lower doses of extrapolated values of <1 TCID_50_ were tested once (*n* = 5 per group) and resulted in 100% survival (Figure 2A). Weight loss also showed a dose-response following IP challenge (Figure 2C). All groups of mice showed some weight loss, even those with 100% survival. Mice challenged with the higher doses lost weight from day 3 onwards but mice challenged with the lower doses did not start losing weight until later. Of the mice that survived, all recovered to their pre-challenge weight within 2 weeks (Figure 2C). The log-rank analysis concluded there was no difference between the dose groups following IP challenge.

Following aerosol challenge of mice with EBOV-Kikwit, there was less of a response compared to IP challenge (Figure 2). For the highest challenge dose, the mean Impinger count was 292 TCID_50_/mL resulting in a retained dose of 9.7 TCID_50_. Only those mice that received the highest retained dose of approximately 10 TCID_50_ showed any lethality or weight loss; three of five mice challenged with 10 TCID_50_ succumbed to infection from day 8 post-challenge (Figure 2B) and showed some weight loss (Figure 2D). Mice challenged with an extrapolated dose of 1, 0.1, or 0.01 TCID_50_ retained dose of EBOV-Kikwit by the aerosol route had 100% survival and no weight loss (Figure 2). The log-rank analysis concluded there was a significant difference in survival between the 10 TCID_50_ group and other groups (*p* = 0.0154) following aerosol challenge.

### 3.3. Combined Historical Data Shows a Range of Lethality with Different EBOV Variants

**Figure 3:**
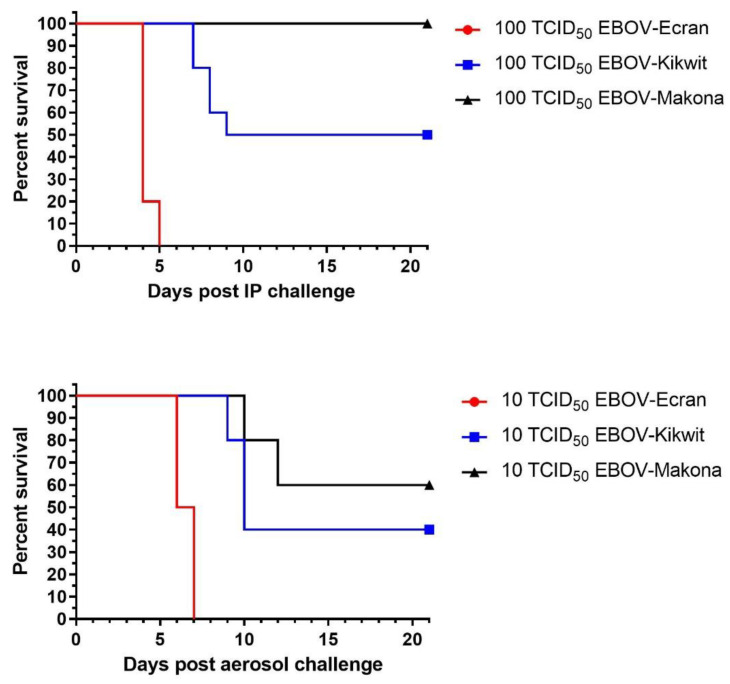
Indicative comparison of the lethality of different EBOV variants. Data from multiple experiments have been combined to give an indication of the difference in survival between EBOV-Ecran (Yambuku variant, red), EBOV-Kikwit (Blue), and EBOV-Makona (black) after a 100 TCID_50_ IP challenge (**top**) or a 10 TCID_50_ retained dose aerosol challenge (**bottom**).

Combining the EBOV-Kikwit data with our previously derived data for EBOV-Ecran (E718, Yambuku 1976 variant) and EBOV-Makona allowed for an indicative comparison of the different variants. This data is indicative because the comparison was not performed in a single experiment. However, the back titer of doses on each individual experiment allowed equivalent doses to be compared in the same immune-deficient mouse model. Doses of 100 TCID_50_ for IP and 10 TCID_50_ for aerosol have been tested for three EBOV variants (Figure 3). For IP challenge, at a dose of 100 TCID_50_, high variability in survival rates was observed ranging from 100% lethality (EBOV-Ecran) to 100% survival (EBOV-Makona). For an aerosol dose of 10 TCID_50_, at this value, all three variants caused some level of lethality (Figure 3). EBOV-Makona only showed lethality by the aerosol route.

A comparison of the three EBOV variants data sets suggests a gradient of lethality with EBOV-Ecran being the most lethal, followed by EBOV-Kikwit then EBOV-Makona. For IP and aerosol challenges and at the doses shown, the log-rank analysis indicated a significant difference between the curves (*p* < 0.0001 for IP and *p* = 0.0002 for aerosol). For both routes, the more virulent variant (EBOV-Ecran) also causes disease quicker with animals taking longer to succumb, if at all, with EBOV-Kikwit and EBOV-Makona.

A direct comparison of MARV-Angola and MARV-Popp within the same experiment has been performed, also with high variability in survival between the variants, but as this has only been done via the IP route and not the aerosol route, data is not included here.

## 4. Discussion

The stability of EBOV and MARV in aerosols was determined to identify if there was a difference in decay rates from viruses isolated from distinct geographical and time periods and to identify any possible correlations between aerosol stability and lethality. Aerosol transmission of filoviruses has been reviewed [22] and the aerosol threat of the Ebola virus or Marburg virus considered [23]. In nature, person-to-person transmission of filoviruses is from contact with contaminated bodily fluids, but infection of animals including non-human primates via the aerosol route is possible in laboratory studies. Additionally, many laboratory methods and hospital procedures [24] can produce aerosols. As aerosol transmission of EBOV or MARV can result in severe disease, an understanding of the stability of filoviruses in aerosols is required to enable modeling in the event of an accidental or deliberate aerosol release.

Currently, the data presented in this paper is the first report on the aerosol stability of EBOV-Kikwit and MARV-Angola (FANG recommended type strains), in an experimentally generated small particle aerosol held under dark conditions, at 20 °C and 50–60% relative humidity. Under the single environmental condition tested, there was no significant difference in the decay rates between MARV-Popp and MARV-Angola or between EBOV-Makona and EBOV-Kikwit with all four viruses being relatively stable with similar decay rates between 1 and 2% min^−1^. Interestingly, although all four viruses had similar starting titers, there was consistently a greater loss of EBOV-Kikwit after aerosolization. The 0 min time-point for EBOV-Kikwit was 10 fold less than for the other viruses. We have previously observed that the titer of impinger counts of EBOV-Kikwit (but not EBOV-Ecran) is affected by freeze-thawing (personal observation), and whilst the samples in this study were titrated by TCID_50_ assay straight away it is possible long-term storage of the virus stock (at −80 °C) made the virus more fragile upon aerosolization. This is a phenomenon that needs more investigation by comparing titers pre and post aerosolization of newly grown virus to virus stored for different periods.

The EBOV data presented in this study proved very different from the only other EBOV isolate comparison published to date. In a 2016 study, the authors compared EBOV-Makona (C07, the same isolate used in this study) and EBOV-Mayinga [25]. Similar to this study, no significant difference between the isolates was reported [25], however, their data suggested the viruses were highly stable reporting the slope of the nonlinear regression to be −0.0029 for their non-normalized data (compared to −0.016 in our study). The previous study estimated it would take over 10 h (619 min) for the virus to reduce in titer by 1 log_10_] [25]. In comparison, the decay rate derived from data in our studies suggests reduction by 1 log_10_ would take 6–7 min. Our study used direct enumeration of the viable virus via TCID_50_ assay whereas the previous study back-calculated the TCID_50_ amount from qRT-PCR data. This, plus other differences in aerosolization methodology and conditions may account for the two highly different data sets.

Multiple factors can affect the survival and stability of viruses in aerosols. It is well known that environmental factors such as temperature and relative humidity as well as sunlight [26,27] and composition of spray material, e.g., [28,29] can affect the stability of micro-organisms in aerosols. For these reasons, studies performed under different conditions at different institutes and using different apparatus are hard to compare. Our data can be compared to itself as the drum experiments were all performed by the same people using the same methodology, and all viruses were grown in the same way. The only variation in the experiments described here is in the virus used.

Our data can be considered a ‘baseline’ of aerosol survival under ambient conditions in the dark. The baseline can then be used to look at the effect of altering various factors (initially one at a time before possibly combining) to see the effect of different aerosol or growth conditions on stability. Some but not all of the factors that could be looked at include a range of temperatures, relative humidity values, and light levels (to simulate sunlight) as well as spraying in the presence of pollutants and other compounds that might be found in the air and looking at different particle sizes. On the virus side, the growth or spray media, the day of harvest, or the passage number might affect stability. There are a huge number of factors to consider, but our data showing minimal variability between different variants/isolates indicates that the work may not need to be done with all, or the most recent virus, but one virus as an exemplar virus for the species.

In terms of consistency, the filovirus decay data reported in this study provide similar decay rates and half-life values to that which we have observed with the Lassa virus [18] and Nipah virus [in preparation]. The same apparatus (same aerosol generation, aerosol conditioning, and particle size), spray conditions, temperature, and relative humidity as well as the same virus enumeration methods were used for all these studies, and all viruses were grown in the same cell line and aerosolized in the same media. The low decay rates we report here for the filoviruses are consistent with data reported by others for the influenza virus and Venezuelan equine encephalitis virus under similar conditions [30,31].

There is minimal data comparing the aerosol stability of different isolates or variants of the same virus, under the same conditions. The stability of different variants and isolates of SARS-CoV-2 within aerosols were reported as being the same irrespective of isolate [32] and is consistent with the stability of the EBOV and MARV variants determined in the present study. This lack of variability in aerosol survival between isolates and variants may be due to the general similarity in chemical structure and composition, which will be similar across variants. It may also be the case that the experimental aerosol apparatus may not be capable of delineating subtle variability between isolates or variants. Minor variations in genetic sequences, may result in more significant changes in characteristics such as virulence and lethality which the interferon mouse may be capable of detecting. Minimal differences in aerosol decay rates for different virus variants may mean that modeling, risk assessments, and control measures will not require changing with every new outbreak or emergence of a new variant or new isolate.

Immune-deficient IFN-α/β R^−/−^ mice have been used to look at the virulence of different species or isolates of filoviruses since this mouse strain was first developed for assessing lethality [33]. The immune-deficient IFN-α/β R^−/−^ mouse is a good model as wild-type virus is used for infection and no time-consuming and potentially virus modifying adaptation of virus is required allowing for rapid testing of new species or variants. We have collated all our data looking at EBOV (aerosol and IP) and MARV (IP only) infection in immune-deficient IFN-α/β R^−/−^ mice and seen a range of responses for both viruses with differences in lethality depending on the variant used. EBOV-Ecran has been shown to be consistently lethal even at very low doses [12] and by injected and aerosol infection routes, whereas EBOV-Kikwit and EBOV-Makona result in reduced mortality in the murine model. In our model, with our stock of EBOV-Makona, this virus only caused lethality by the aerosol route [13].

The virulence of different EBOV variants was reviewed recently [34] and showed that whilst EBOV-Mayinga (highly conserved in terms of sequence identity to EBOV-Ecran), EBOV-Kikwit, and EBOV-Makona all cause 100% lethality in non-human primates or ferrets at low doses, the murine model was able to identify differences in virulence and infectivity as we have seen. The data with EBOV-Kikwit reported here are similar to those reported previously [33,35]. Early work compared the 1976 and 1995 variants of EBOV in the IFN-α/β R^−/−^ mouse; the EBOV from 1976 (equivalent to our EBOV-Ecran) resulted in 100% lethality, whilst the “EBO-Z 95” (a previous name for the EBOV-Kikwit variant) resulted in 100% survival after injected challenge [33]. Brannan et al showed that doses of 1000–5000 PFU of EBOV-Kikwit, given via the IP route, resulted in weight loss and some lethality, but all doses had 80% or greater survival [35]. Data presented here adds to our knowledge with further doses of injected EBOV-Kikwit and aerosol challenge data, confirming the lower levels of lethality compared to those observed with EBOV from 1976.

Whilst the mouse data presented here with EBOV-Kikwit is consistent with previously reported data, the lethality of EBOV-Makona in the murine models may be more complex. Previously we reported findings of 100% survival following injected challenge with the C07 isolate of the EBOV-Makona variant [13]. Similar findings of 100% survival but weight loss and signs of disease with recovery were also reported after a range of different EBOV-Makona isolates were tested [36]. However, a recombinant- EBOV-Makona C07 resulted in 100% mortality with a dose of 1 PFU [37] and the mouse-adapted EBOV-Makona is also 100% lethal at 10 and 1 PFU challenge doses [38]. The variability of virulence of EBOV-Makona in mice may reflect the number of different EBOV-Makona isolates that are available from different times and geographical locations of the West Africa outbreak and the difference between using wild-type virus versus recombinant or adapted virus.

In our murine model, the gradient of virulence-associated with three EBOV variants is Ecran (Yambuku variant, 1976) most virulent, followed by Kikwit (1995) and then Makona (2013) as least virulent. This gradient does match the severity of those disease outbreaks if ranked by overall case fatality rate (Table 1), [4,5]) but this also follows a chronological decrease which may be expected with reporting, diagnostics, and treatment during human outbreaks improving over time. Case fatality rates in human outbreaks are also affected by more than just the causative agent with geographical, socio-economic, political, and cultural factors having an impact [39].

Investigations using both the murine model and aerosol decay in the Goldberg drum could be extended into other EBOV or MARV isolates or different variants if available. Based on our observations presented here, we would predict that we might continue to see variability in the virulence of EBOV variants in our murine model, but similar survival of different filovirus isolates in aerosols.

In conclusion, the immune-deficient mouse model proved to be a useful model for the discrimination of different lethality rates associated with different filoviruses. Therefore, for rapidly responding and characterizing new isolates or variants, the immune-deficient mouse may prove to be the best first step. Evidence presented in this study suggests that aerosol stability is less likely to differ with new isolates or variants.

## Figures and Tables

**Table 1:** Summary of Ebola virus and Marburg virus types discussed in this paper.

Virus	Ebola Virus			Marburg Virus	
Variant-Isolate used in this work	Yambuku-Ecran	13625-Kikwit	Makona-C07	Hesse-Poppinga	Angola-200501379
Name in this paper	EBOV-Ecran	EBOV-Kikwit	EBOV-Makona	MARV-Popp	MARV-Angola
Country of origin	Zaire	Zaire	Guinea ^1^	Uganda ^2^	Angola
Year of outbreak	1976	1995	2013–2016	1967	2004–2005
No. fatalities/Total No. cases	280/318	245/317	11,325/28,652	7/31	227/252
Case fatality rate	88%	77%	40%	23%	90%
Passage used	4	3	6	8	4
Stock titre (TCID_50_/mL)	1 × 10^7^	1 × 10^7^	8 × 10^6^	2 × 10^7^	1 × 10^7^
Illumina MiSeq sequencing top hit (NCBI Reference No.)	Ebola virus/H.sapiens-tc/COD/1976/Yambuku-Mayinga (NC_002549)	Ebola virus/H.sapiens-tc/COD/1995/Kikwit-807223 (KR063672)	Ebola virus/H.sapiens-wt/GIN/2014/Makona-C05 (KT013255)	n/d, partial sequencing and PCR confirmation only	Marburg virus/H.sapiens-tc/AGO/2005/Angola-200501379 (KR867677)

^1^ Spread to France, Germany, Italy, Liberia, Mali, Netherlands, Nigeria, Norway, Senegal, Sierra Leone, Spain, Switzerland, UK and USA; ^2^ Spread to West Germany and Yugoslavia.

## Data Availability

© Crown copyright (2022), Dstl. This material is licensed under the terms of the Open Government Licence except where otherwise stated. To view this licence, visit http://www.nationalarchives.gov.uk/doc/open-government-licence/version/3 (accessed on 6 April 2022) or write to the Information Policy Team, The National Archives, Kew, London TW9 4DU, or email: psi@nationalarchives.gov.uk.
